# Distribution, Removal, and Risk Assessment of Pharmaceuticals and Their Metabolites in Five Sewage Plants

**DOI:** 10.3390/ijerph16234729

**Published:** 2019-11-27

**Authors:** Ying Li, Xiangming Niu, Chi Yao, Wen Yang, Guanghua Lu

**Affiliations:** Key Laboratory of Integrated Regulation and Resource Development on Shallow Lake of Ministry of Education, College of Environment, Hohai University, Nanjing 210098, China; hj6688@hhu.edu.cn (Y.L.); 181805010004@hhu.edu.cn (X.N.); yaochi@hhu.edu.cn (C.Y.); 181305020038@hhu.edu.cn (W.Y.)

**Keywords:** pharmaceuticals and personal care products, sewage treatment plant, removal efficiency, mass balance, risk assessment

## Abstract

The extensive use of pharmaceuticals and personal care products (PPCPs) leads to a continuous increase of their presence in urban wastewater. These pollutants are discharged into natural waters and pose a threat to human health and the ecological environment. This study focused on five sewage treatment plants in three cities of China’s Yangtze River Delta as research sites to study the distribution and degradation of drugs and their conversion products in wastewater. The concentration of target compounds in the water ranged from 0 to 510.8 ng/L, and both positive and negative removal rates occurred during the treatment. Acetaminophen (ACE) and ibuprofen (IPF) can be completely removed in the biological treatment stage. The addition of flocculants and sand filtration has a positive effect on the removal of naproxen (NPX) and bezafibrate (BZB). Ultraviolet disinfection is beneficial for the removal of antipyrine (ATP) and diclofenac (DCF). A small amount of PPCPs were found in the sludge and particulate matter, which had little effect on removal. Finally, the risk quotients were used to evaluate the harmfulness of the PPCPs detected in the effluent to the ecological environment, and the results showed that there was little hazard.

## 1. Introduction 

With the rapid development of the social economy, an increasing number of types of pharmaceuticals and personal care products (PPCPs) have been formulated. Because PPCPs are widely available, easily accessible, convenient and effective in use, their presence continues to increase in the environment. Today, the presence of PPCPs has been detected in wastewater, surface water, and groundwater at the levels of ng^−1^ to μg^−1^ [1,2,3]. When the concentration of PPCPs in the environment reaches a certain level, it will be transmitted to the human body through drinking water, the food chain, and other ways. Finally, it will cause harm to human body. 

Previous studies [4] have shown that PPCPs, such as tetracycline and triclosan, can damage the photosystem II reaction center of algae by oxidative stress and inhibit the synthesis of some proteins and chloroplast formation. Trace PPCPs in water can affect the growth and reproduction of microorganisms. They affect the normal growth of plants and reduce the content of cytochrome and green leaves. This leads to the increase of the deformity rate of animals and the occurrence of growth and reproduction obstacles. In addition, some substances will be transformed into other substances when they are discharged into water. These products are even more toxic than the parent compounds [5]. Therefore, it is very important to study the occurrence, fate, and effects of pharmaceuticals and their metabolites in the environment.

Sewage treatment plant (STP) is the source and sink of pharmaceuticals, and it is of interest to study the occurrence and fate of pharmaceuticals in different treatment processes of STP to understand their behavior, effects, and loading in receiving aquatic environments. Relevant research [6] has shown that pharmaceuticals removal is related to the treatment temperature, redox conditions, chemical conditions, and hydraulic retention time of the STP. Some organic compounds flowing into STPs can almost completely degrade in the treatment process, such as acetaminophen and ibuprofen [7], and some drugs are partially degraded (diclofenac, carbamazepine, etc.) [8]. There are some drugs with higher concentrations in the effluent than in the influent. This may be due to the release of organic matter adsorbed in particulate matter during the treatment process or the conversion of conjugated metabolites in the matrix by enzymatic reaction [9]. PPCPs have been widely examined in aquatic environment, but little research has focused on their metabolites and removal efficiencies in different treatment stages of STP.

In this study, the degradation of diclofenac, naproxen, and seven other pharmaceuticals in STPs was discussed. The objectives of this study were (1) to investigate the occurrence, distribution, and fate of PPCPs and their metabolites through the different treatment stages of STPs in the Yangtze River Delta, (2) to research the effects of different wastewater treatment processes on the removal of PPCPs and their metabolites, and (3) to evaluate the ecological risk of the release of these PPCPs and their metabolites into the receiving river.

## 2. Experimental

### 2.1. Chemicals and Methods

The purity of all standards used in this study exceeded 99%. Antipyrine (ATP) and naproxen (NPX) were purchased from Sigma Aldrich Trading Co., Ltd. (Shanghai, China). Acetaminophen (ACE) was purchased from CND Isotopes (Quebec, QC, Canada). Bezafibrate (BZB), 4-chlorobenzoicacid (4-CBA), ibuprofen (IPF), clofibric acid (CA), diclofenac (DCF), and nifedipine (NP) were purchased from Bailingwei Technology Co., Ltd. (Beijing, China). ACE-d3, ATP-d3, BZB-d6, CA-d4, DCF-d4, and IPF-d3 were purchased from Toronto Research Chemicals (Toronto, ON, Canada). Chromatographic grade methanol, acetonitrile, and acetone were purchased from Merck (Darmstadt, Germany). Selected target compounds and their related properties are shown in Table 1.

### 2.2. Sample Collection

This study selected five STPs located in China’s Yangtze River Delta region: Lianxi Sewage Treatment Plant (Plant A), Beilaowei Sewage Treatment Plant (Plant B), Kuncheng Photoelectric Water Purification Company (Plant C), Kunshan Sewage Treatment Plant (Plant D), and Nanjing Tiebei Sewage Treatment Plant (Plant E). Plant A and Plant B are located in Hefei City. Plant C and Plant D are located in Kunshan, and Plant E is located in Nanjing. Plant B is located in an industrial park, and most of the influent water is industrial sewage. The sequencing batch reactor activated sludge process (SBR) and Fenton oxidation are mainly used in this STP. Plant D uses two processes, anoxic/oxic (A/O) and circulating activated sludge treatment (CAST), and the effluent is mixed and discharged finally. A small portion of the wastewater of Plant E comes from an industrial park. The specific sewage treatment processes are provided in the supplementary information (Figure S1).

**Figure 1:**
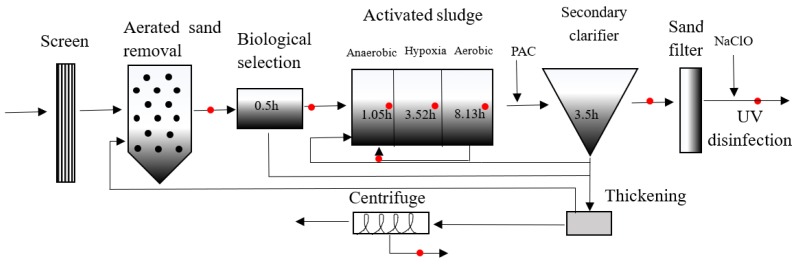
Sampling points in the Kuncheng Photoelectric Water Quality Purification Company.

Sampling points were set at each processing section in each STP, and activated sludge was taken from the STPs. Sampling points of Plant C are shown in Figure 1, and the others are listed in Table S1 and Figure S1. From April to June 2019, the first stage of sampling was carried out. At the end of each treatment process, 4 L of a 24 h mixed water sample were collected using a 2 L stainless steel sampling bucket and stored in a brown polyethylene bottle. The water sample was stored at 4 °C and treated within 48 h. The activated sludge was collected and stored in a self-sealing polyethylene bag and placed in a refrigerator at −20 °C prior to further processing. 

### 2.3. Sample Preparation

The pretreatment of the water samples was carried out by solid phase extraction (SPE). First, a water sample was filtered using a 0.45 μm glass fiber filter, and the filter was stored for analysis of the particulate matter. Second, 1000 mL of the filtered water sample was put into a glass sample bottle, and 100 μL of internal standard (mixed standard of 6 internal standards, 1.0 mg/L) was added. The target drugs in the water sample were then enriched with an oasis hydrophilic–lipophilic balance (HLB) solid phase extraction column. The activated sludge sample and the suspended particulate matter were treated following methods described previously [10]. Details about the treatment procedures of the samples are provided in the supplementary information Section S1.1.

### 2.4. Analytical Protocol

The prepared sample extracts were analyzed by liquid chromatography/tandem mass spectrometry. The chromatographic separation was performed on a US Waters ACQUITY ultra high-performance liquid chromatograph (UPLC). The column was a Waters BEH C18 column (2.1 × 100 mm, 1.7 μm), and the column temperature was 40 °C. The target drugs were separated by gradient elution. The mobile phases for positive ion mode (ESI+) included Mobile Phase A (98% water and 2% methanol containing 0.05% formic acid) and Mobile Phase B (acetonitrile). The mobile phases for negative ion mode (ESI−) were the same as those implemented in positive ion mode. The flow rate was set to 0.4 mL/min, and the injection volume was 5 μL. The detailed instrumental analysis methods are described in the supplementary information, Section S1.2, Tables S2 and S3. 

### 2.5. Quality Assurance and Quality Control

Six points in the concentration range from 1.0 to 200.0 μg/L were selected for the generation of calibration curves, and the correlation coefficients were all above 0.99. The 10-fold signal-to-noise ratio (S/N = 10) of the blank sample was used as the limit of quantitation, and 3 times the signal-to-noise ratio (S/N = 3) was the limit of detection (Table S4). The method recoveries were calculated. Quality assurance and quality control (QA/QC) samples were analyzed the same way as the samples. Ultra-pure water was used for the recovery test, and the recovery rates ranged from 71 to 126%. The results show that the recovery rate of the water samples was 40–117%, and the recovery rate of sludge was 40–112%, which is consistent with the conclusions obtained in previous experiments [6,11]. Lower recovery rates may be associated with complex matrices [11,12,13,14], as signal suppression was observed for most analytes. The recovery rates of all analytes are shown in Tables S5 and S6.

### 2.6. Calculation of Mass Loads and Removal Efficiency

We calculated the daily mass load of each analyte in the STPs for mass balance analysis. We use the following formula to calculate the daily mass load for each analyte [15]:

(1) $$M_{influent}=C_{influent}\times Q_{sewage}$$

(2) $$M_{effluent}=C_{effluent}\times Q_{sewage}$$

(3) $$M_{sluge}=C_{sluge}\times Q_{sludge}\;$$

where *M_influent_* is the daily mass load of the respective compound, *M_effluent_* is the mass load in the treated effluent, and *M_sludge_* is the daily mass load in sludge. *C_influent_*, *C_effluent_*, and *C_sludge_* are the concentrations of each analyte in the influent, effluent, and sludge, respectively. *Q_sewage_* and *Q_sludge_* are the daily flow of the sewage plant and the daily production of sludge, respectively.

The mass load was calculated by multiplying the measured concentration of each analyte by the flow per day. We used Equation (4) to calculate the total loss of compounds in STPs [15]:

(4) $$\mathrm{Total}\;\mathrm{loss}\left(\%\right)=\frac{M_{influent}-M_{effluent}-M_{sludge}-M_{particulates}}{M_{influent}}\times 100\%$$

where *M_particulates_* is the mass load in daily particulate matter.

The removal efficiency of PPCPs in each wastewater treatment unit and the average removal rate of PPCPs after sewage treatment were calculated using Equation (5):

(5) $$\mathrm{Removal}\;\mathrm{rate}\left(\%\right)=\frac{C_{influent}-C_{effluent}}{C_{influent}}\times 100\%.$$

## 3. Results and Discussion

### 3.1. Occurrence of PPCPs at Different Sewage Treatment Plants

Some drugs are very effective in treating common diseases, and non-prescription drugs are widely available. Every year, a large number of drugs are used by people. Some drugs that are not absorbed by the human body are discharged into water bodies with urine and feces. Some drugs are difficult to degrade in natural water, leading to continuous increases in PPCPs in water. Therefore, PPCPs can be detected in municipal domestic sewage plants. In the study of the five STPs in this work, the target compounds were detected in different processing sections. The highest concentration was several hundred nanograms. Some target compounds were still detected in the effluent. The sewage treatment process achieved the complete removal of some organic compounds. The removal rates of ACE, NPX, and IPF were 100%. However, some organic compounds showed negative removal. For example, DCF was enriched in the process of water treatment. After analyzing the effects of various processes on the removal of PPCPs, it was found that the biological treatment stage could not only decompose some organic compounds (ACE, etc.) but also increase the concentration of some precursor compounds. The addition of flocculant and ultraviolet disinfection were beneficial to the removal of organic compounds.

#### 3.1.1. Distribution of PPCPs in the Influent of Sewage Treatment Plants

As Table 2 shows, all drugs and their metabolites were detected among the five STPs in China’s Yangtze River Delta region. In Plant A, 4-CBA, CA, and NP were not detected, the concentration of ACE was as high as 511.3 ng/L likely because acetaminophen is the most commonly used drug to relieve pain and fever, and it is estimated that billions of doses of acetaminophen are annually consumed [16]. The concentration of ACE in this study (511.3 ng/L in Plant A, 208.0 ng/L in Plant C, and 510.8 ng/L in Plant E) is similar to that previously reported (350 ng/L) by Al-Odaini et al. [17]. However, only ATP, DCF, and BZB were detected in Plant B, the concentrations of ATP, DCF, and BZB were 5.8, 5.7, and 0.8 ng/L, respectively. The concentrations of DCF in these STPs were lower than that reported by Koutsouba [18] and Kosma [9] in Greek sewage plants (up to 560 ng/L and average 2.0 μg/L, respectively). The frequency of drug use in different regions and the metabolism of drugs in the human body may be the factors leading to the differences. In addition, different external factors (light, temperature, etc.) may also cause this phenomenon. Plant B is located in an industrial park of Hefei, and most of the influent is industrial sewage. It contains fewer PPCPs, as only a small part of domestic sewage containing PPCPs flows into Plant B. In Plant C, we found that the concentration distribution of nine PPCPs ranged from the highest, with ACE at 208 ng/L, to the lowest, with CA at 2.2 ng/L, and NP and NPX were not detected. The analysis of Plant D showed that ACE, IPF, CA, and NP were not detected. Among the remaining PPCPs, 4-CBA was the highest (88.0 ng/L), and ATP was the lowest (1.2 ng/L). In the influent water of Plant E, only CA and NPX were not detected, and the concentration ranged from 1.1 (ATP) to 510.8 ng/L (ACE). From Table 2, the concentration distribution of IPF ranges from 21.8 ng/L in Plant A to 49.6 ng/L in Plant C, the concentrations were all at even nanogram level. Wiegel et al. [19] reported that the concentration of IPF in the influent of a sewage plant in Germany was 0.03 μg/L. Similar results were obtained in our work. The concentration of BZB found in this experiment is low (<4.1 ng/L), but the findings of Christina, Verlich, and Niina are very high, which are 210.4, 20.0, and 53.0 ng/L, respectively [6,20,21]. This may be the reason for the frequency of drug use. Table S7 shows that the detection rates of antipyretic analgesics and anti-inflammatory drugs (51.3%) and their concentrations were relatively high. The detection rate of hypolipidemic drugs was low. The NP detection rate of antihypertensive drugs was only 22.5% (the lowest), which may be related to the type of drugs used in the region.

#### 3.1.2. Distribution of PPCPs in the Effluent of Sewage Treatment Plants

In previous studies [7,17], it has been noted that some PPCPs can be removed by a sewage treatment process. In the present study, it can be seen from Table 2 that the concentration of some PPCPs was greatly reduced relative to that in the influent. At Plant A, we found that IPF, BZB, and ACE detected in the influent were not detected in the effluent. This is consistent with the findings of other researchers [8,9], because IPF, BZB, and ACE have good degradation in sewage treatment process. It is worth noting that CA (2.6 ng/L) was present in the effluent but was not detected in the influent. Emma [7] found a similar situation in study. This may be due to the presence of glucuronide and other conjugated metabolites. Only ATP and BZB were detected in the effluent of Plant B, with concentrations of 2.4 and 1.1 ng/L, respectively. 4-CBA, DCF, and BZB were detected in the effluent of Plant C. The effluent of Plant D is the confluence of two different water treatment processes, with more drugs detected. After analyzing the effluent from Plant E, it was found that the concentration of DCF increased significantly. Compared with that of the influent, the concentration of some drugs was shown to decrease significantly, indicating that organic compounds were degraded in the process of water treatment. However, there were also some drugs that tended to increase in concentration and may have even been undetectable in the influent. This may be related to the water solubility, adsorption, and degradation conditions of the drugs or sampling errors caused by long hydraulic residence times. The remaining concentration of the detected drugs in the effluent are outlined in Table 2.

#### 3.1.3. Distribution of PPCPs in Sludge and Suspended Particles

The collected sludge and filter membrane were ultrasonically extracted, and it was found that only DCF, 4-CBA, NPX, and CA were detected among the five sewage plants (Table 2). This is also related to their higher K_ow_. It is also possible that the complexes formed by the addition of Fe ions in the sewage increases the aggregation–flocculation of the organic compounds [22]. The content of drugs in the particulate of this experiment was low, so it was not included in the mass load.

### 3.2. Mass Loads and Mass Balance Analysis

**Figure 2:**
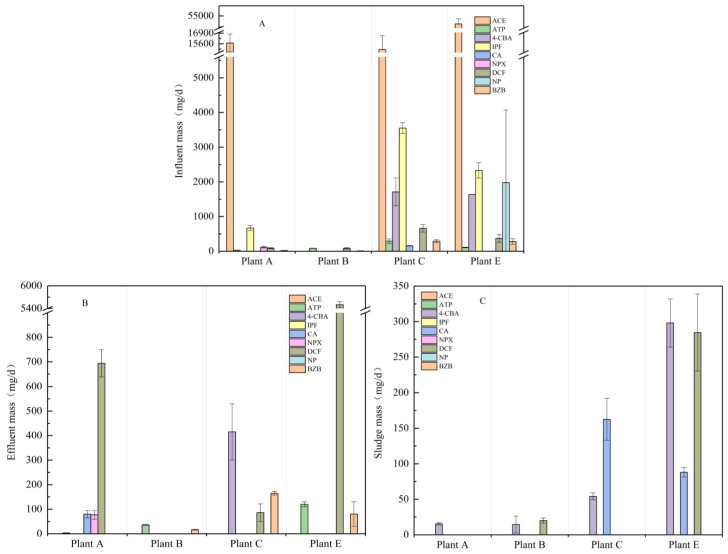
Influent load (**A**), effluent load (**B**), and sludge load (**C**) of PPCPs in five sewage treatment plants.

To evaluate the input and output of PPCPs in each STP, we use Equations (1)–(3) to calculate the mass loads of the target drugs. Figure 2 and Table 3 show the daily mass load of the target drugs in each STP. The average load of a single drug entering an STP ranged from 3.1 to 51,080 mg/day. Antipyretic analgesics (ACE) and anti-inflammatory drugs (IPF and DCF) are loaded in water at a high level of several milligrams. The daily mass load of ACE was on the level of tens of grams. The highest ACE mass load observed was 51.1 g/day, which was also in direct proportion with the high frequency use of the precursor drug in daily life. Other drugs in the water were relatively low. Although ACE and IPF have a large influent load, the better removal of ACE and IPF can reduce the impact on the surrounding ecosystem. On the contrary, the negative removal of DCF leads to the increase of effluent load, which has a potential threat to the surrounding ecosystem. To study the fate of common PPCPs, total mass loss (Equation (4)) was used to calculate the mass balance. Table 3 shows that the total mass loss of ACE, NP, and IPF was 100%. It was not found in the sludge, indicating that it was completely biodegraded. The total mass loss of ATP ranged from −9 to 89%. The total mass loss of BZB ranged from −38 to 100%. The total mass loss of ATP in Plant E and that of BZB in Plant B were negative, but positive removal was found in the process of treatment. The concentration of the final effluent is higher than that of the influent, leading to negative removal. The total mass loss of NPX was 34%. These compounds were not found in the sludge. However, 4-CBA (in Plant A and Plant B) and CA (in Plant B and Plant E) were found in sludge but were not found in water. This may be because the concentration of the drug in the water was below the detection limit, and the sludge has a long-term adsorption effect. From Table 3, the removal capacity of the STP to the target material presents an excellent and extremely bad trend. The removal rate of some drugs (ACE, IPF, NP, etc.) was very high (100%), and some drugs (DCF and ATP) showed negative removal.

### 3.3. Removal Efficiencies of Different Sewage Treatment Plants 

#### 3.3.1. Removal Rate in Sewage Treatment Plant

Some organic compounds will decompose under certain natural light conditions. In an experiment of DCF irradiation by ultraviolet light, Li et al. [23] found that six kinds of conversion products were produced. Some drugs have high degradation efficiency in the process of biodegradation. For example, both ACE and IPF have good biodegradability [17], and the degradation efficiency can reach 100% [7]. In addition, the removal of drugs may be related to the water treatment process, temperature, pH, hydraulic retention time, and the added chemicals in the process of sewage treatment.

**Figure 3:**
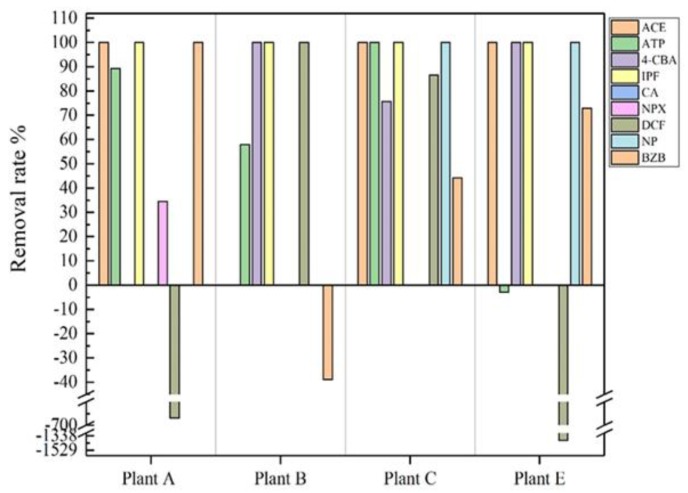
Average removal rate of PPCPs after sewage treatment (the result is calculated from Equation (5). *C_influent_* represents the concentration of PPCPs in the influent of an STP, and *C_effluent_* represents the concentration of PPCPs in the effluent of an STP).

Table 4 shows the removal rate of the target drugs in each sewage treatment unit, and Figure 3 shows the removal rate of the target drugs after the whole sewage treatment process. Figure 3 shows that the removal rates of ACE, antipyretic, and analgesic drugs were all 100%. Because ACE has good biodegradability in water, it can be completely removed in the biological treatment stage of A^2^/O (anaerobic-anoxic-oxic). Lin et al. [24] compared the single adsorption and adsorption–biodegradation tests of ACE. They found that biodegradation is the main degradation mode of ACE, and the effect of adsorption is almost non-existent. The removal rate of ATP ranged from 0 to 100% (Figure 3) in the effluent. ATP may be difficult to biodegrade and may easily decompose under light conditions. Previous reports [25,26] show that the degradation efficiency of ATP by traditional sewage treatment is relatively low (about 30%). However, ATP degradation based on photodegradation has a good effect [27,28]. The detection rate of lipid-lowering drugs (BZB) was high (83.7%, Table S7), while the detection rates of CA and 4-CBA were only 38.1 and 32.6%, respectively. The removal rates of 4-CBA were 76–100%, and the removal rates of BZB were −39–100% (Figure 3). We found that the concentration of BZB in the effluent from Plant B increased after treatment. This may be due to the release of BZB in the form of glucuronides in water [29] that cleaved in an SBR (styrene butadiene rubber)–Fenton process. IPF can be completely degraded during the sewage treatment process mainly because it is easy to photolysis, has a high biodegradation rate, and the effect of an added flocculant iron ion. Naproxen was detected only in Plants A and D, and 35% was removed in Plant A and 100% in Plant D. The lower removal efficiency of NPX may be attributed to its persistence under microbial attack. In addition, NPX is easy to react with free chlorine, so disinfection may be a major factor leading to higher removal [30]. This unbalanced removal efficiency has also been found in European countries—for example, 66% in Germany [31], 40–55% in Spain [32], and 94% in Sweden [33]. DCF not only exhibited positive removal but also showed negative removal in these STPs. DCF is a highly hydrophilic organic compound. Solid phase adsorption has little effect on it. The removal of DCF mainly depends on biodegradation [8]. However, Bo et al. [34] found that the biodegradability of DCF was difficult to confirm. At present, there is no accurate conclusion. It may be that precursor compounds are continuously released from glucuronic acid and other conjugated metabolites. At the same time, Cl– and N–H functional groups inhibit the growth of microorganisms in the process of biodegradation, resulting in negative removal. Therefore, negative removal has been found in many studies [35,36].

#### 3.3.2. Removal Efficiency in the Biological Treatment Unit

The STPs in this study are largely based on A^2^/O. Previous studies [17,37] have found that ACE is easily degradable at the biological treatment stage. ACE can be used as the sole carbon source to metabolize and decompose in the stage of biological treatment. Table 4 shows that ACE did not exist in water after anaerobic treatment in the present study. We can conclude that anaerobic treatment is an effective way to degrade ACE. In the STPs studied, ATP in the anaerobic and anoxic stages showed a decreasing trend but remained almost unchanged in the aerobic stage. This may be due to the re-release of the anaerobic and anoxic adsorbed particles. We found that 4-CBA, CA, IPF, NPX, and BZB were degraded in the anoxic phase. The degradation of IPF was best in the aerobic phase, but BZB showed decreasing removal. The DCF content increased during the anaerobic and anoxic aerobic stages. This may be due to the enzymatic processes of aerobic treatment, which results in the conversion of glucuronic acid and other metabolites back into the precursor compounds [35]. In addition, the change of samples caused by the release of substances adsorbed on the particles and the longer hydraulic retention time may also be the influencing factors [9].

#### 3.3.3. Removal Efficiency of the Precipitation, Filtration, and Ultraviolet Disinfection Process

Precipitation, filtration, and ultraviolet disinfection processes can also effectively remove PPCPs [8,22]. ATP easily decomposes under the condition of light. The bonds of N–N and C–N on ATP were easily attacked and broken, and different series of photodegradation intermediates were then produced by demethylation and bond recombination. Plant A, Plant C, and Plant E use ultraviolet light to disinfect the effluent. Table 4 shows that the relative removal efficiency of ATP was 43–100%. However, ATP was not effectively removed in the effluents of Plant B and Plant D without ultraviolet disinfection (as seen in the untreated effluent samples). It was indicated that ultraviolet disinfection is the main removal method of antipyrine in sewage. The removal of 4-CBA in both the sedimentation tank and the filtration process may be due to the adsorption of particulate matter. The addition of flocculant may also lead to the increase of the trend of the complex formed with iron ions, thus promoting the removal. The concentration of NPX increased in the inclined plate filter and sedimentation tank, which may be the result of the re-release of organic matter adsorbed to the particulate matter. After sand filtration, the concentration decreased again, which is in accordance with the results published by Ziylan and Ince [8]. This shows that sand filtration is beneficial to the removal of NPX. In the process of sewage treatment, a certain amount of flocculant is added to accelerate precipitation, which also explains the slight decrease in the DCF concentration. Kosma et al. [9] previously reported that the disinfection process can play a positive role in the removal of DCF. In this study, the removal rate of Plant B and Plant C was very obvious (>99%), which is also confirmed by the experimental conclusion. However, it was not removed (−4%) in Plant E, which may be caused by too much negative removal during anaerobic treatment [6].

## 4. Risk Assessment of PPCPs in Effluent

**Figure 4:**
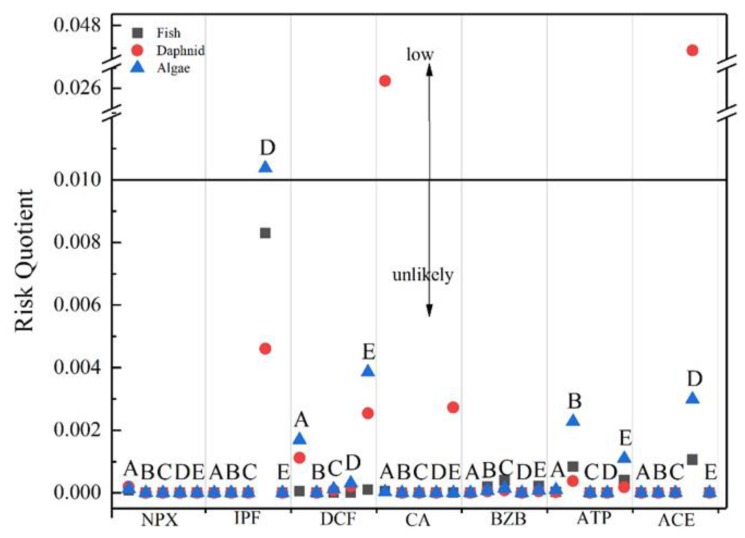
Environmental risk assessment results for the detected drugs in the effluent.

Studies have shown that trace amounts of PPCPs can cause direct or indirect toxicity to aquatic organisms and human health. Therefore, it is essential to assess the risk of PPCPs in the water to the surrounding ecosystem. However, it is difficult to estimate whether pharmaceuticals will adversely affect non-target organisms at the environmental level. Hazard quotients are an effective way to characterize the potential risks of PPCPs [38], according to the relevant documents of the European Union on environmental risk assessment. The risk quotient method was used to evaluate the environmental risk level of the PPCPs in the effluent of the five STPs in this study and was calculated using Equation (6) [39]:

(6) $$\mathrm{RQ}=\frac{MEC}{PNEC}$$

where MEC represents the actual measured maximum concentration, and PNEC is the predicted no-effect concentration, which was obtained from the literature [39,40,41,42,43]. 4-CBA and NP temporarily lack corresponding data, so they were not evaluated in this study. In fact, owing to the lack of chronic toxicity data, the effective evaluation of drugs often meets this major obstacle. The EC50 value is used to predict PNEC, thereby assessing the adverse effects of the detected drug concentration on aquatic organisms. Three trophic levels (fish, daphnid, and algae) were selected in the aquatic ecosystem to calculate the RQ value (Figure 4). There is a potential high risk when the calculated RQ > 1, a medium risk when 1 > RQ > 0.1, and a low or negligible risk when 0.1 > RQ > 0.01 [36,44,45,46]. By analyzing the calculated results, only the RQ of CA detected in Plants A and D and IPF detected in Plant D were between 0.1 and 0.01, indicating a low and almost negligible hazard. The other calculated RQ values were less than 0.01.

**Figure 5:**
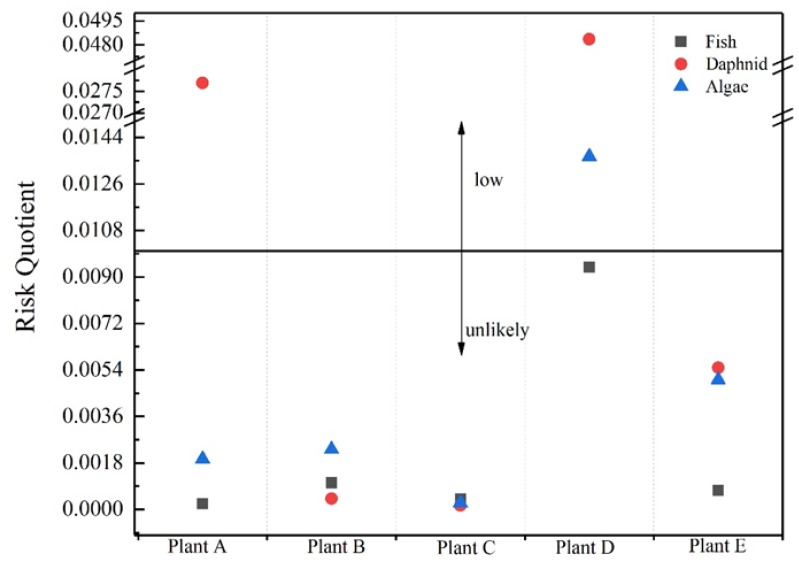
The sum of the risk quotients of compounds in each STP.

Many studies have evaluated the effects of a single compound on aquatic organisms. However, many compounds have synergistic effects when combined. In this experiment, the target organic compounds have a similar mode of action. Therefore, it was assumed that the effect of the mixture follows the concentration increase model. The RQ of each evaluation point is the sum of the RQ values of the individual compounds. Figure 5 shows that the RQ of daphnid in Plant A was 0.027. The RQ of daphnid in Plant D was 0.048, and the RQ value of algae was 0.013. All of these RQ values indicate a low risk. 

## 5. Conclusions

In this study, we studied the occurrence and distribution of PPCPs in five STPs. The highest concentration was 511.3 ng/L (ACE) in the influent of Plant A. The lowest concentration was 0.1 ng/L (ATP) in the effluent of Plant A, and the mean detection rate of PPCPs was 51.3%. PPCPs in wastewater can only be partially removed in the STP processes. Biodegradation is the main method of removal for these organic compounds. The adsorption of the compounds to sludge and suspended particulate matter and the addition of flocculants and ultraviolet disinfection only played an auxiliary role. In the process of biological treatment, some target drug (ATP and DCF) concentrations appeared to be negatively removed. This may be from adsorption and desorption or the conversion of other compounds. The risk quotients of PPCPs in the effluent were calculated to evaluate the environmental risk level. It can be concluded that only Plant A and Plant D show low risks, and the wastewater discharge from the other STPs had little impact on the ecological environment.

## Figures and Tables

**Table 1:** Selected target compounds and their related properties.

Compounds	Molecular Formula	Physicochemical Properties	Use
Antipyrine ATP CAS:60-80-0	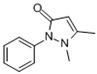	MW: 188.23 Log Kow: 0.59 WS: 1000 g/L	Antipyretic, analgesic
Naproxen NPX CAS:22204-53-1	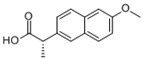	MW: 230.26 Log Kow: 2.5	Antipyretic, analgesic
Acetaminophen ACE * CAS:130-90-2	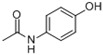	MW: 151.16 Log Kow: 0.27 WS: 14 g/L	Antipyretic, analgesic
4-Chlorobenzoic acid 4-CBA * CAS:74-11-3	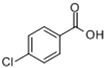	MW: 156.57 Log Kow: 2.52	Hypolipidemic
Ibuprofen IPF CAS:15687-27-1	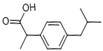	MW: 206.28 Log Kow: 3.79	Anti-inflammatory
Clofibric acid CA CAS:882-09-7	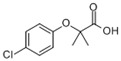	MW: 214.64 Log Kow: 2.84	Hypolipidemic
Nifedipine NP CAS:21829-25-4	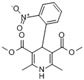	MW: 346.34 Log Kow: 2.50	Antihypertensive
Bezafibrate BZB CAS:41859-67-0	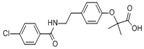	MW: 361.83 Log Kow: 4.25	Hypolipidemic
Diclofenac DCF CAS:15307-86-5	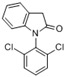	MW: 296.15 Log Kow: 4.02	Anti-inflammatory, analgesic

4-CBA is the transformation product of BZB, and ACE is the transformation product of phenacetin. * indicates a transformation product.

**Table 2:** The concentration of PPCPs in the sludge, particulate, influent, and effluent.

PPCPs		ACE	ATP	4-CBA	IPF	CA	NPX	DCF	NP	BZB
Plant A	Influent (ng/L)	511.3 ± 35.5	0.9 ± 0.1	-	21.8 ± 2.5	-	3.8 ± 0.8	2.9 ± 0.4	-	0.65 ± 0.07
	Effluent (ng/L)	-	0.1 ± 0.0	-	-	2.6 ± 0.5	2.5 ± 0.6	22.6 ± 1.8	-	-
	Sludge (ng/g)	-	-	0.9 ± 0.1	-	-	-	-	-	-
	Particulate (ng/g)	-	-	14.5 ± 9.5	-	3.0 ± 0.3	-	-	-	-
Plant B	Influent (ng/L)	-	5.8 ± 0.3	-	-	-	-	5.7 ± 1.3	-	0.8 ± 0.1
	Effluent (ng/L)	-	2.4 ± 0.1	-	-	-	-	-	-	1.1 ± 0.1
	Sludge (ng/g)	-	-	2.3 ± 1.9	-	-	-	3.2 ± 0.6	-	-
	Particulate (ng/g)	-	-	29.1 ± 1.2	-	-	-	14.6 ± 1.9	-	-
Plant C	Influent (ng/L)	208 ± 23.9	4.1 ± 0.9	23.9 ± 5.6	49.6 ± 2.2	2.2 ± 0.1	-	9.2 ± 1.5	-	4.1 ± 0.6
	Effluent (ng/L)	-	-	5.8 ± 1.6	-	-	-	1.2 ± 0.5	-	2.3 ± 0.1
	Sludge (ng/g)	-	-	1.1 ± 0.1	-	3.3 ± 0.6	-	-	-	-
	Particulate (ng/g)	-	-	12.8 ± 4.2	-	-	-	10.0 ± 0.8	-	-
Plant D	Influent (ng/L)	-	1.2 ± 0.1	88.0 ± 4.7	-	-	4.3 ± 2.4	61.2 ± 2.9	-	2.2 ± 0.3
	Effluent (ng/L)	360.2 ± 35.1	1.9 ± 0.2	4.1 ± 0.7	38.6 ± 2.8	-	-	4.1 ± 0.3	13.7 ± 2.1	-
	Particulate (ng/g)	-	-	11.2 ± 2.1	-	-	80.5 ± 23.8	6.6 ± 0.8	-	-
Plant E	Influent(ng/L)	510.8 ± 25.3	1.1 ± 0.1	16.4 ± 0	23.3 ± 2.2	-	-	3.7 ± 1.1	19.8 ± 20.9	2.8 ± 0.8
	Effluent (ng/L)	-	1.2 ± 0.1	-	-	0.3 ± 0.1	-	55 ± 0.8	-	0.8 ± 0.5
	Sludge (ng/g)	-	-	4.4 ± 0.5	-	1.3 ± 0.1	-	4.2 ± 0.8	-	-
	Particulate (ng/g)	-	-	35.5 ± 9.5	-	-	-	5.2 ± 0.8	-	-

**Table 3:** The daily mass load, mass loss, and total mass loss of target substances in each sewage treatment plant.

	PPCPs	ACE	ATP	4-CBA	IPF	CA	NPX	DCF	NP	BZB
Plant A	Influent (mg/d)	15,696.9	27.6	-	669.3	-	116.7	89.0	-	20.0
	Effluent (mg/d)	-	3.1	-	-	79.8	76.8	693.8	-	-
	Sludge (mg/d)	-	-	15.4	-	-	-	-	-	-
	Mass loss (mg/L)	15,696.9	24.6	−15.4	669.3	−79.8	39.9	−604.8	-	20.0
	Total mass loss	100%	89%	-	100%	-	34%	−679%	-	100%
Plant B	Influent (mg/d)	-	87.0	-	-	-	-	85.5	-	12.0
	Effluent (mg/d)	-	36.0	-	-	-	-	-	-	16.5
	Sludge (mg/d)	-	-	14.4	-	-	-	20.0	-	-
	Mass loss (mg/L)	-	51.0	−14.4	-	-	-	65.5	-	−4.5
	Total mass loss	-	59%	-	-	-	-	77%	-	−38%
Plant C	Influent (mg/d)	14,887.0	293.4	1710.6	3550.0	157.5	-	658.5	-	293.4
	Effluent (mg/d)	-	-	415.1	-	-	-	85.9	-	164.6
	Sludge (mg/d)	-	-	54.2	-	162.5	-	-	-	-
	Mass loss (mg/L)	14,887.0	293.4	1241.3	3550.0	−5.1	-	572.6	-	128.8
	Total mass loss	100%	100%	73%	100%	−3%	-	87%	-	44%
Plant E	Influent (mg/d)	51,080.0	110.0	1640.0	2330.0	-	-	370.0	1980.0	280.0
	Effluent (mg/d)	-	120.0	-	-	-	-	5500.0	-	80.0
	Sludge (mg/d)	-	-	298.1	-	88.1	-	284.6	-	-
	Mass loss (mg/L)	51,080.0	−10.0	1341.9	2330.0	−88.1	-	−5414.6	1980.0	200.0
	Total mass loss	100%	−9%	82%	100%	-	-	−1463%	100%	71%

**Table 4:** The removal rate of PPCPs in each sewage treatment unit.

STP	Treat Process	ACE	ATP	4-CBA	IPF	CA	NPX	DCF	NP	BZB
Plant A	Anaerobic	−11%	−89%	-	−31%	-	−273%	−130%	-	−264%
	Hypoxic	100%	−134%	-	100%	-	30%	−1442%	-	70%
	Aerobic	-	1%	-	-	23%	21%	8%	-	−29%
	Secondary clarifier	-	2%	-	-	−4%	−4%	10%	-	−4%
	Inclined plate clarifier	-	17%	-	-	28%	−11%	72%	-	236%
	Effluent	100%	97%	-	100%	−24%	73%	6%	100%	100%
Plant B	Chemical reaction cell	-	−3%	-	-	-	-	61%	-	−61%
	Air flotation tank	-	−1%	-	100%	-	-	−123%	-	32%
	SBR	-	18%	-	-	-	-	−169%	-	−124%
	Fenton	-	47%	-	-	-	-	69%	-	50%
	Triple settling tank	-	15%	100%	-	-	-	−11%	-	7%
	Effluent	-	−11%	-	-	-	-	100%	-	−23%
Plant C	Anaerobic	−43%	−11%	−5%	−200%	−25%	-	−627%	-	34%
	Hypoxic	96%	−39%	62%	59%	22%	-	−18%	20%	2%
	Aerobic	100%	−55%	100%	100%	0%	-	−4%	100%	−5%
	Secondary clarifier	-	30%	-	-	−5%	-	−4%	-	18%
	Inclined plate clarifier	-	−20%	2%	-	−16%	-	−9%	-	3%
	Effluent	-	100%	33%	-	16%	-	99%	-	−3%
Plant D	Anaerobic/Hypoxic	-	−183%	−374%	-	-	100%	37%	-	19%
	Aeration	100%	−79%	68%	−9%	-	-	−92%	7%	−33%
	Grate	-	−129%	13%	79%	-	-	−12%	100%	21%
	Anaerobic	98%	−36%	−397%	−58%	-	100%	−1112%	-	45%
	Aerobic	100%	−9%	23%	100%	-	-	−20%	-	100%
	Secondary clarifier	-	−40%	55%	-	-	−16%	0%	100%	-
	Effluent	-	0%	-	-	-	8%	-	-	-
Plant E	Anaerobic	37%	−24%	−187%	−283%	-	-	−1228%	−236%	4%
	Hypoxic	100%	−50%	100%	100%	-	-	−34%	−9%	85%
	Aerobic	-	2%	-	-	-	-	−1%	77%	−88%
	Secondary clarifier	-	3%	13%	-	-	-	−1%	100%	2%
	Sand filter	-	−2%	100%	-	-	-	20%	-	−18%
	Effluent	-	43%	-	-	-	-	−4%	-	12%

## Supplementary Materials

The following are available online at https://www.mdpi.com/1660-4601/16/23/4729/s1. Table S1. Information about sewage plant. Table S2. The composition of two ion mode mobile phase. Table S3. The mass spectrometer optimization parameters of target compounds. Table S4. Limit of detection and limit of quantitation of target substances. Table S5. The recovery rate of all analytes in water. Table S6. The recovery rate of all analytes in sludge and particulars. Table S7. Detection rate of various drugs in various sewage treatment plants. Table S8. Risk assessment of different PPCPs against different aquatic species. Figure S1. Flow chart of treatment process in sewage treatment plant.
