# Supplementary material for: Distribution, Removal, and Risk Assessment of Pharmaceuticals and Their Metabolites in Five Sewage Plants
Source: Int J Environ Res Public Health. 2019 Nov 27;16(23):4729. doi: 10.3390/ijerph16234729 (PMC6926846; doi:10.3390/ijerph16234729)
Supplement: Supplementary file 1 [file ijerph-16-04729-s001.pdf]

Distribution, removal, and risk assessment of pharmaceuticals and their metabolites in five sewage plants

Ying Li, Xiangming Niu, Chi Yao, Wen Yang, Guanghua Lu*

Key Laboratory of Integrated Regulation and Resource Development on Shallow Lake of Ministry of Education, College of Environment, HoHai University, Nanjing, 210098, China

Supplementary Information:

1 Materials and methods

1.1 Sampling preparation

The pretreatment of the water samples was carried out by solid phase extraction (SPE). First, a water sample was filtered using a 0.45 μm glass fibre filter, and the filter was stored for analysis of the particulate matter. Second, 1000 mL of the filtered water sample was put into a glass sample bottle, and 100 μL of internal standard (mixed standard of 6 internal standards, 1.0 mg/L) was added. Then, the target drugs in the water sample were enriched with an oasis hydrophilic-lipophilic balance (HLB) solid phase extraction column. The activated sludge sample and the suspended particulate matter were treated following the methods described by Wan et al.

The extraction column was activated with 5 mL of methanol and ultrapure water before enrichment. After the extraction was completed, the column was washed with 10 mL of ultrapure water, and vacuum conditions were continued for 30 min to remove the water from the column. The column was then eluted with 10 mL of methanol, which was collected in a 15 mL glass tube. The eluent was evaporated under nitrogen to 100 μL, resuspended to 1 mL with methanol, filtered through a 0.22 μm organic filter and stored in a 2 mL brown sample vial at -20 °C prior to analysis.

The appropriate amount of Na_2HPO_4, Na_2EDTA and citric acid were used to prepare 0.2 mol/L solutions, and the prepared Na_2HPO_4 solution and the citric acid solution were mixed at a volume ratio of 8:5 to prepare a McIlvaine solution. Then, the McIlvaine solution and the Na_2EDTA solution were mixed in a volume ratio of 1:1 to prepare a 0.1 mol/L EDTA-McIlvaine mixed solution, and the pH was adjusted to 4 using HCl. Methanol, acetonitrile and acetone were mixed at a volume ratio of 2:2:1 to prepare an organic mixed extract, which was adjusted to pH 4 with H_3PO_4.

After lyophilization, the activated sludge samples were ground and sieved (2 mm). Then, 1 g of the solid sample and 10 mL of EDTA-McIlvaine buffer were subjected to shaking for 30 s, and then 50 μL (1.0 mg/L) internal standard was added. The samples were centrifuged at 4500 r/min for 15 min, the supernatant was transferred to a brown container, and the extraction was repeated twice more in the same manner with the organic mixed extract. The extracts were combined, degreased with 10 mL of n-hexane, diluted to 500 mL with ultrapure water, passed through a 0.45 μm fibre filter, adjusted to pH 4 with H_3PO_4, and then loaded at a rate of 3 to 5 mL/min onto HLB columns. After sample loading, the HLB column was rinsed with 10 mL of ultrapure water and vacuum-dried for 30 min. Finally, the columns were eluted with 10 mL of methanol, and the eluents were evaporated to near dryness under a nitrogen flow. Samples were reconstituted to 1 mL, filtered through 0.22 μm filters and analysed.

1.2 Analytical protocol

The prepared sample extracts were analysed by liquid chromatography/tandem mass spectrometry. The chromatographic separation was performed on a US Waters ACQUITY ultra high-performance liquid chromatograph (UPLC). The column was a Waters BEH C18 column (2.1×100 mm, 1.7 μm), and the column temperature was 40 °C. The target drugs were separated by gradient elution. The mobile phases for positive ion mode (ESI+) included mobile phase A (98% water and 2% methanol containing 0.05% formic acid) and mobile phase B (acetonitrile). The mobile phases for negative ion mode (ESI-) were the same as those implemented in positive ion mode. The flow rate was set to 0.4 mL/min, and the injection volume was 5 μL.

The mobile phase gradient is described in Table S1. Mass spectrometry was performed using a Waters ACQUITY XevoTQ with an ESI source set to 150 °C. The acquisition method was multiple reaction monitoring (MRM) mode. The atomized desolvation gas and collision gas were high purity nitrogen and high purity argon at flow rates of 900 L/h and 0.15 mL/min, respectively. The capillary voltage was 3.0 kV, the temperature of desolvation gas was 500 °C, and the cone backflush gas flow rate was 50 L/h. ACE and ATP were detected in positive ion mode, while 4-CBA, IPF, CA, DCF, NPX, NP, and BZB were detected in negative ion mode. The precursor ions, the product ions, the collision voltages, and the collision energies of the target drugs are shown in Table S2.

1.3 Data analysis

Statistical analysis was performed using Microsoft Excel 2016 and Origin 2017 software. Means and

standard deviations were calculated from triplicate measurements.

Lianxi Sewage Treatment Plant

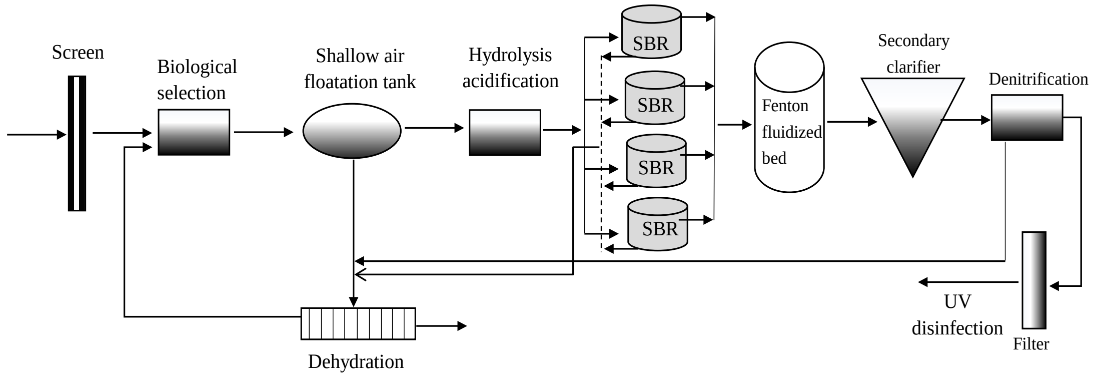

Beiliao Wastewater Treatment Plant in Binhu New Area

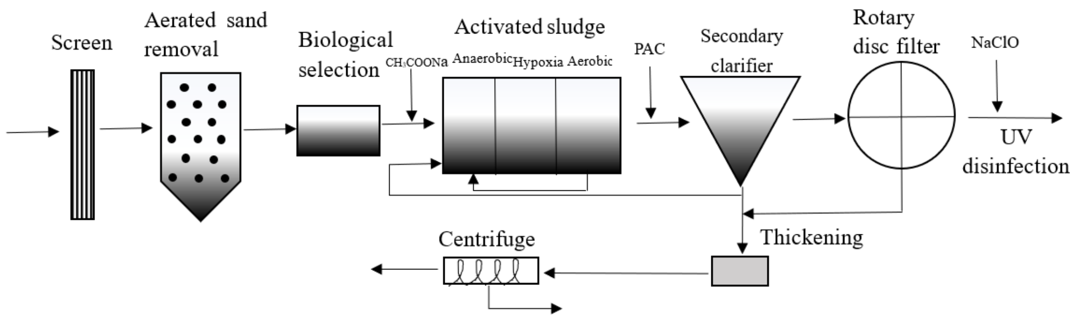

Kuncheng Photoelectric Plant

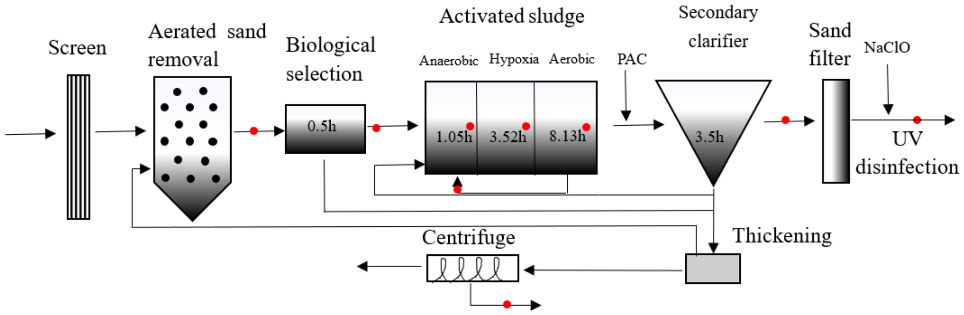

Kunshan Sewage Treatment Plant

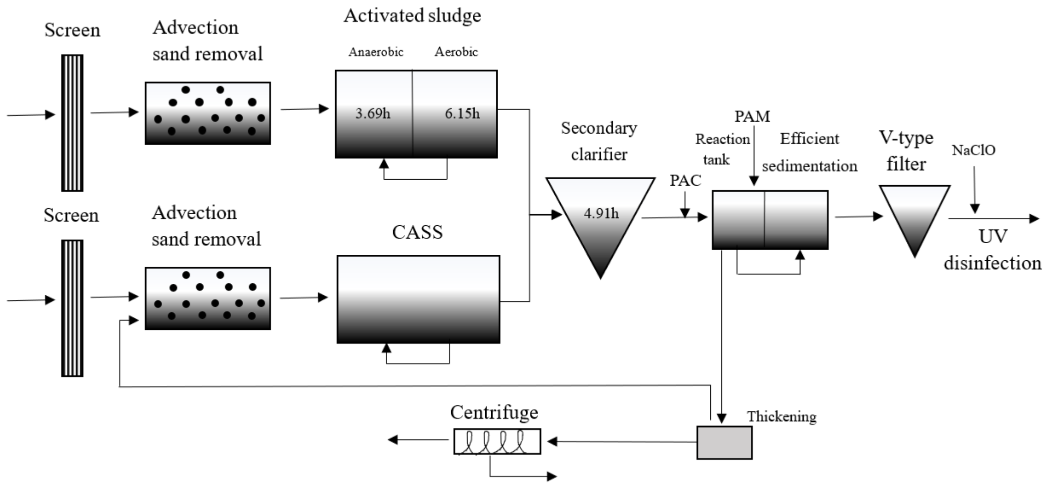

Nanjing Tiebei Sewage Treatment Plant

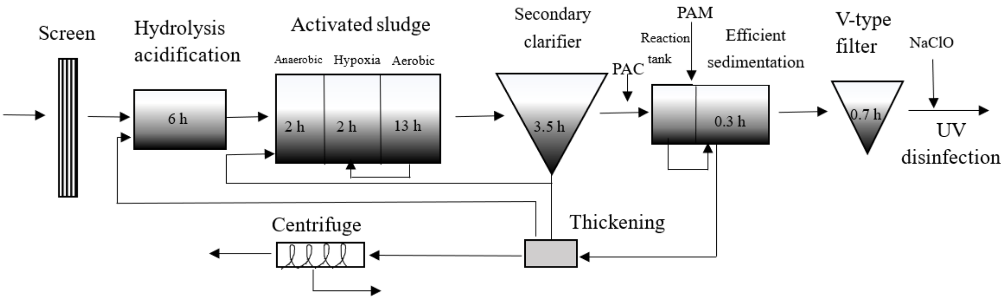

Figure S1. Flow chart of treatment process in sewage treatment plant

Table S1. Information about sewage plant

STP	type	Daily water load (thousand tons)	Daily sludge load (kg)	Main process
Plant A	municipal	30.70	17082	A^2O
Plant B	municipal, industrial	15.00	6240	SBR, Fenton oxidation
Plant C	municipal	71.57	49257	A^2O
Plant D	municipal	-	-	A/O, CAST
Plant E	municipal	100.00	67760	A^2O

Table S2. The composition of two ion mode mobile phase

Time（min）	Mobile phase composition %	
ESI+	A	B
0	90	10
2.5	90	10
4	10	90
5	10	90
5.01	90	10
6	90	10
ESI-	A	B
0	90	10
2.5	90	10
4	10	90
5	10	90
5.01	90	10
6	90	10

Table S3. The mass spectrometer optimization parameters of target compounds

PPCPs	Parent ion (m/z)	Subion ion (m/z)	Collision voltage (V)	Collision energy (eV)	Pattern
ACE	152	110	40	10	ESI+
ACE-d_3	155	92.9	26	22	ESI+
ATP	189	76.8	40	32	ESI+
ATP-d_3	192	58.9	40	28	ESI+
4-CBA	157	113	24	10	ESI-
IPF	205.1	205.1	16	2	ESI-
IPF-d_3	208.1	164	16	7	ESI-
CA	213.1	127	24	16	ESI-
CA-d_4	217.2	131	20	18	ESI-
NPX	229.1	169.9	18	14	ESI-
NPX-d_3	232.1	173	22	16	ESI-
DCF	194	214	22	22	ESI-
DCF-d_4	298	254	22	15	ESI-
NP	345	122	22	12	ESI-
BZB	360	274	32	22	ESI-
BZB-d_6	366.3	274.1	30	18	ESI-

Table S4. Limit of detection and limit of quantitation of target substances

Compounds	LOQ (ng/L)	LOD (ng/L)
ACE	1.360	0.408
ATP	0.164	0.049
4-CBA	6.667	2.000
IPF	1.399	0.420
CA	0.067	0.020
NPX	0.740	0.222
DCF	0.173	0.052
NP	0.240	0.072
BZB	0.081	0.024

LOD refer to limits of detection (ng/L) that were determined as lowest concentration corresponding to the signal-to-noise(S/N) ratio of 3.

LOQ refer to limits of quantification (ng/L) that were determined as lowest concentration corresponding to the signal-to-noise(S/N) ratio of 10.

Table S5. The recovery rate of all analytes in water

Analyte	Ultrapure water								Wastewater													
	Original (ng/L)	50ng/L						Recovery	Original (ng/L)						50ng/L							Recovery
		1	2	3	Mean (ng/L）	SD	RSD %		1	2	3	Mean (ng/L）	SD	RSD %	1	2	3	SD	Mean (ng/L）	SD	RSD %	
ACE	-	40.2	39.4	38.9	39.50	0.66	1.66	79%	115.7	94.1	97.0	102.3	11.7	11.5	131.8	116.4	126.7	7.8	125.0	7.8	6.28	45%
ATP	-	56.3	55.4	55.1	55.60	0.62	1.12	111%	0.8	0.7	0.7	0.7	0.1	7.9	32.7	31.7	33.1	0.7	32.5	0.72	2.22	64%
4-CBA	-	59.6	62.6	60.2	60.80	1.59	2.61	122%	39.3	-	30.5	34.9	6.2	17.8	105.6	85.3	73.9	16.1	88.3	16.06	18.19	107%
IPF	-	29.5	41.2	35.7	35.47	5.85	16.50	71%	20.6	9.1	12.5	14.1	5.9	42.0	30.8	34.3	38.7	4.0	34.6	3.96	11.44	41%
CA	-	59.8	57.1	51.5	56.13	4.23	7.54	112%	-	-	-	-	-	-	27.8	24.3	26.2	1.8	26.1	1.75	6.71	52%
NPX	-	53.5	61	68.4	60.97	7.45	12.22	122%	-	-	-	-	-	-	40.7	30.9	35.9	4.9	35.8	4.90	13.68	72%
DCF	-	35.9	39.9	44.1	39.97	4.10	10.26	80%	31.0	31.6	32.4	31.7	0.7	2.2	60.1	60.2	60.7	0.3	60.3	0.32	0.53	57%
NP	-	38.5	40.2	37.3	38.67	1.46	3.77	77%	-	-	-	-	-	-	59.5	55.4	60.4	18.4	58.4	2.67	4.56	117%
BZB	-	65.2	60.8	63.7	63.23	2.24	3.54	126%	0.3	0.7	0.3	0.4	0.2	53.3	23.4	20.4	23.3	1.7	22.4	1.70	7.62	44%

Table S6. The recovery rate of all analytes in sludge and particulars

Analyte	Original						50ng/g						Recovery
	1	2	3	Mean（ng/L)	SD	RSD	1	2	3	Mean（ng/L)	SD	RSD	
4-CBA	18.7	21.8	27.5	22.67	4.46	20%	82.7	75.5	63.7	73.97	9.59	13%	103%
IPF	-	-	-	-	-	-	15.9	25.9	18	19.93	5.27	26%	40%
CA	6.7	8.8	8.4	7.97	1.12	14%	35.3	32.4	32.7	33.47	1.59	5%	51%
NPX	7.6	11.5	2.8	7.30	4.36	60%	36.7	28.5	31.7	32.30	4.13	13%	50%
DCF	-	-	-	-	-	-	26.4	33.6	25.9	28.63	4.31	15%	57%
NP	15.6	31.3	15	20.63	9.24	45%	94.9	71.9	63.6	76.80	16.22	21%	112%
BZB	5.6	5.4	5	5.33	0.31	6%	32.8	25.8	29.6	29.40	3.50	12%	48%

Table S7. Detection rate of various drugs in various sewage treatment plants

	plant A	plant B	plant C	plant D	plant E	Average
ACE	42.9%	0.0%	42.9%	40.0%	14.3%	28.0%
ATP	100.0%	100.0%	85.7%	100.0%	100.0%	97.1%
4-CBA	0.0%	14.3%	47.6%	86.7%	14.3%	32.6%
IPF	42.9%	9.5%	42.9%	53.3%	28.6%	35.4%
CA	100.0%	0.0%	76.2%	0.0%	14.3%	38.1%
NPX	100.0%	0.0%	0.0%	33.3%	0.0%	26.7%
DCF	100.0%	86.7%	100.0%	100.0%	100.0%	97.3%
NP	14.3%	0.0%	14.3%	26.7%	57.2%	22.5%
BZB	66.7%	100.0%	100.0%	53.3%	98.6%	83.7%
Average	63.0%	34.5%	56.6%	54.8%	47.5%	51.3%

Table S8. Risk assessment of different PPCPs against different aquatic species

PPCPs	Plant	Fish		Daphnid		Algae		Reference
		EC_{50}(mg/L)	RQ	EC_{50}(mg/L)	RQ	EC_{50}(mg/L)	RQ	
	A		0.00009		0.00019		0.00013	
	B		-		-		-	
NPX	C	34	-	15	-	22	-	Sanderson et al.
	D		-		-		-	
	E		-		-		-	
	A		-		-		-	
	B		-		-		-	Sanderson et al.
IPF	C	5	-	9.02	-	4	-	Lee et al.
	D		0.00830		0.00460		0.01038	Stauer-Lauridsen et al.
	E		-		-		-	
	A		0.00005		0.00112		0.00170	
	B		-		-		-	
DCF	C	532	0.00000	22	0.00008	14.5	0.00012	Grung et al.
	D		0.00001		0.00020		0.00030	
	E		0.00011		0.00254		0.00386	
	A		0.00005		0.02636		0.00003	
	B		-		-		-	
CA	C	53	-	0.11	-	86	-	Hernando et al.
	D		-		-		-	
	E		0.00001		0.00273		0.00000	
	A		-		-		-	
	B		0.00020		0.00004		0.00007	
BZB	C	6	0.00040	30	0.00008	18	0.00013	Hernando et al.
	D		-		-		-	
	E		0.00022		0.00004		0.00007	
	A		0.00002		0.000003		0.00007	
	B		0.0004		0.00007		0.002	
ATP	C	5.781	-	36.797	-	1.346	-	Sanderson et al.
	D		-		-		-	
	E		0.0002		0.00003		0.0009	
	A		-		-		-	
	B		-		-		-	
ACE	C	378	-	9.2	-	134	-	Grung et al.
	D		0.00106		0.04355		0.00299	
	E		-		-		-	

Reference

Grung M, Kallqvist T, Sakshaug S, Skurtveit S, Thomas KV. Environmental assessment of Norwegian priority pharmaceuticals based on the EMEA guideline. Ecotoxicology and Environmental Safety 2008; 71(2): 328-340.

Hernando MD, Aguera A, Fernandez-Alba AR. LC-MS analysis and environmental risk of lipid regulators. Analytical and Bioanalytical Chemistry 2007; 387(4): 1269-1285.

Lee Y, Lee SE, Lee DS, Kim YH. Risk assessment of human antibiotics in Korean aquatic environment. Environmental Toxicology and Pharmacology 2008; 26 (2): 216-222.

Sanderson H, Johnson DJ, Wilson CJ, Brain RA, Solomon KR. Probabilistic hazard assessment of environmentally occurring pharmaceuticals toxicity to fish, daphnids and algae by ECOSAR screening. Toxicology Letters 2003; 144 (3): 383-395.

Stauer-Lauridsen F, Birkved M, Hansen LP, Holten Lützhoft HC, Halling-Sorensen B. Environmental risk assessment of human pharmaceuticals in Denmark after normal therapeutic use. Chemosphere 2000; 40 (7): 783-793.

Weining, W.; Xi, C.; Haimei, J. Simultaneous Determination of Multiple Antibiotic Residues in Livestock Manure by Solid Phase Extraction-Ultra Performance Liquid Chromatography Tandem Mass Spectrometry. Chinese J Anal Chem. 2013, 41, 993-999.
